# Multi-objective optimization of low-noise pervious concrete using a stacking ensemble learning and NSGA-II approach

**DOI:** 10.1038/s41598-026-54224-6

**Published:** 2026-06-11

**Authors:** Fan Yu, Haoyun Zhang, SiYe Zhang, Yang Zhang, Jie Liu, Mingjun Hu

**Affiliations:** 1 College of Civil Engineering and Architecture, China Three Gorges University, Yichang, 443002 China; 2 Key Laboratory of Geological Hazards on Three Gorges Reservoir Area, Ministry of Education, Three Gorges University, Yichang, 443002 China; 3 Hubei Hongchang Architectural Decoration Engineering Co., Ltd., Wuhan, 430064 China; 4 Faculty of Infrastructure Engineering, Dalian University of Technology, Dalian, 116024 China

**Keywords:** Pervious concrete, Sound absorption performance, Multi-objective optimization, Stacking ensemble learning, NSGA-II algorithm, Engineering, Materials science, Mathematics and computing

## Abstract

The inherent performance conflicts among the acoustic, mechanical, and hydraulic properties of pervious concrete represent a core obstacle to its application as a low-noise pavement material. To address this challenge, this paper proposes a multi-objective synergistic optimization method based on Stacking ensemble learning and the NSGA-II algorithm to proactively optimize mix proportions, thereby achieving a balance and enhancement of multiple performance metrics. A comprehensive database, comprising both proprietary experimental data and data from the literature, was first established to systematically train and construct a high-precision predictive model for the sound absorption performance of pervious concrete. Subsequently, this model was combined with previously established models for compressive strength and permeability to serve as the fitness functions for the NSGA-II genetic algorithm, which performed a multi-objective search for optima. The accuracy and reliability of the optimization results were then confirmed through experimental validation. Results indicate that aggregate gradation has a significant impact on the sound absorption of pervious concrete, with a relative performance improvement of 95.7% between the optimal and poorest gradations. The constructed Stacking ensemble learning model achieved a coefficient of determination (R^2^) of 0.97, outperforming all individual models with minimal fluctuation. The proposed multi-objective optimization framework successfully resolved the intrinsic conflict between permeability, compressive strength, and sound absorption. The optimized mix proportion solution (O3) not only satisfied the standards for permeability and strength but also achieved superior sound absorption performance that surpassed all single-sized aggregate groups, with an error of only 8.9% between the model’s prediction and the experimental value.

## Introduction

 The rapid acceleration of global urbanization, coupled with a surge in traffic volume and higher vehicle speeds, has led to increasingly severe noise pollution. This issue has emerged as a critical environmental challenge affecting the quality of life for urban residents^1,2^. To address the growing problem of traffic noise, the development and application of environmentally friendly and functional low-noise pavement materials have become a research hotspot and a key development direction in the field of road engineering.

Pervious concrete (PC) is a sustainable material used in sponge city infrastructure, notable for its interconnected pore network (15–30% porosity) that ensures high permeability^3–5^. This same porous structure also allows PC to act as an effective sound absorber, much like traditional fibrous materials^6–10^. The sound attenuation process begins when traffic-generated sound waves enter the pavement’s pores. Inside, the acoustic energy is dissipated and converted into heat through a combination of viscous losses, thermal conduction, and friction between the oscillating air and the pore walls, thereby leading to a substantial reduction in overall traffic noise^11–13^.

It is well-established that the sound absorption performance of pervious concrete was governed by its internal pore structure, defined by parameters such as porosity, pore size, connectivity, and tortuosity^14–16^. These characteristics were intrinsically linked to the geometry and gradation of the aggregate, as well as the cement paste content^17,18^. Porosity was considered the most decisive factor. The majority of studies demonstrated a strong positive correlation between porosity and sound absorption, as a more porous structure offers extended pathways for sound wave propagation and energy dissipation through conversion to heat^14,19–23^. Other parameters were also significant. Smaller aggregate sizes at similar porosities could refine the pore network for better absorption^24,25^, while optimized aggregate gradation enhanced pore connectivity and tortuosity^26^. The cement paste volume, which controlled porosity, presented a fundamental trade-off, as reducing it to improve acoustic properties can be detrimental to the material’s compressive strength^27^. Consequently, while there is a consensus on enhancing acoustic performance through pore structure optimization, this approach creates inherent conflicts with other essential material properties. The central challenge in the field, therefore, is to systematically resolve the trade-offs between acoustic absorption, permeability, and compressive strength. Developing a methodology to maximize sound attenuation without compromising the necessary permeability and strength of pervious concrete is a critical research gap that needs to be addressed.

**Fig. 1:**
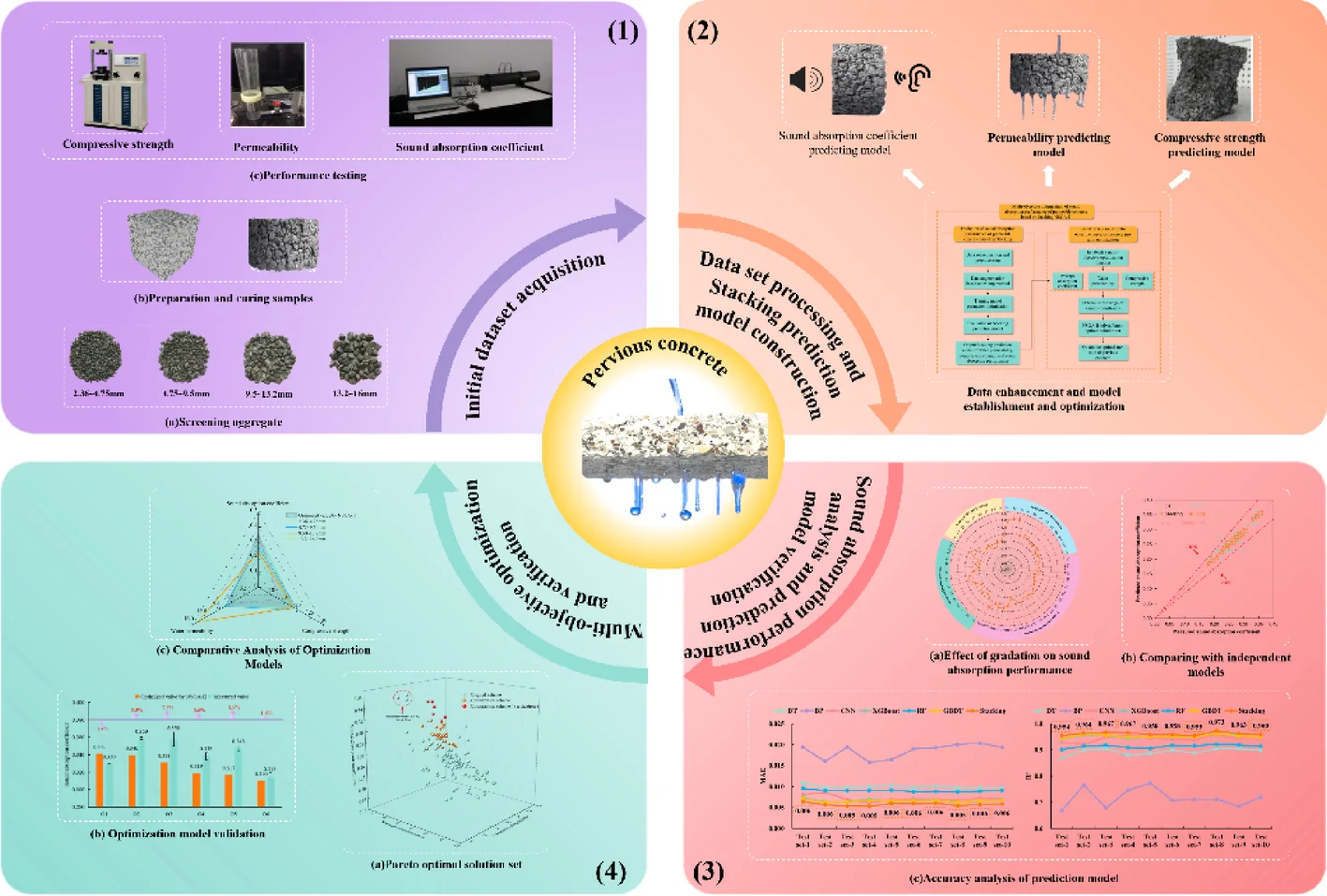
The flow chart of this paper.

Therefore, this paper proposed a multi-objective optimization method based on Stacking ensemble learning and the NSGA-II genetic algorithm to develop pervious concrete with superior acoustic, permeability, and mechanical properties. First, a high-precision Stacking model was specifically constructed and trained to predict the sound absorption coefficient of pervious concrete. This model, in conjunction with previously established predictive models for compressive strength and permeability coefficient from our prior research, was integrated as the fitness functions for the NSGA-II algorithm. The algorithm was then employed to perform a multi-objective search for optimal mix proportions, aiming to obtain a set of Pareto optimal solutions that balance these key performance indicators. Finally, the accuracy and feasibility of the optimization results were confirmed through experimental validation (Fig. 1).

## Methodology

### Data collection and preprocessing

The predictive performance and generalization capability of a machine learning model are fundamentally dependent on the scale, quality, and diversity of the underlying dataset. To ensure a comprehensive basis for our model, the dataset employed in this research was compiled from two distinct sources: a proprietary dataset generated through our own experiments, and a supplementary dataset collected from previously published literature.

#### Proprietary dataset

**Fig. 2:**
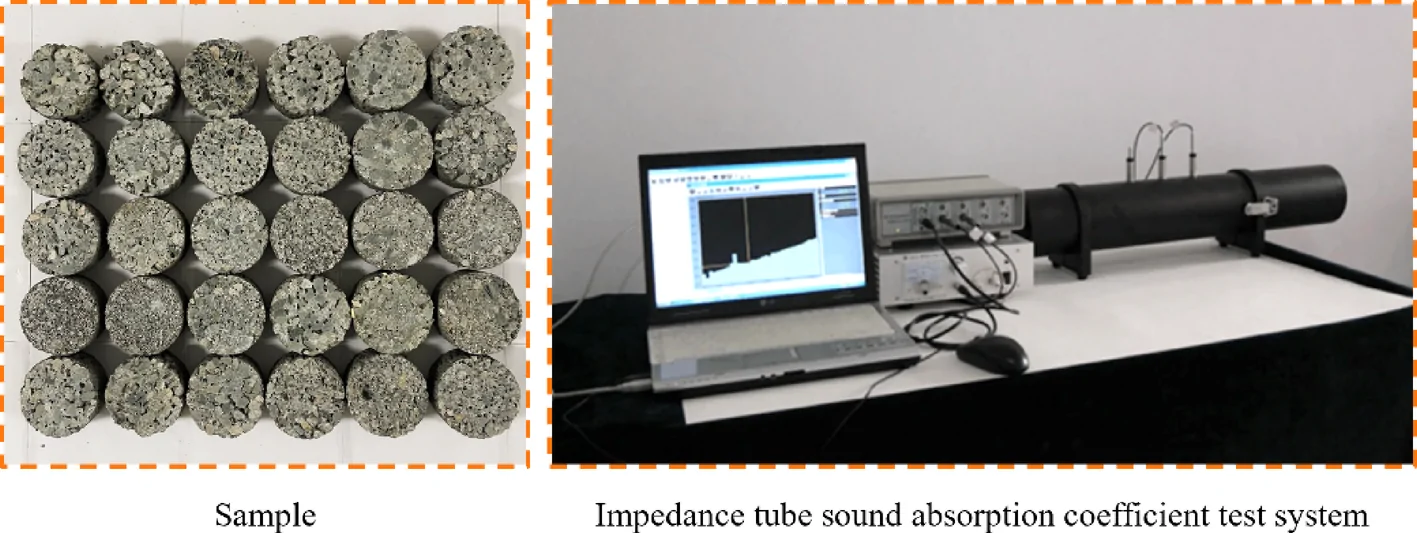
Impedance tube sound absorption coefficient test system.

To construct a representative dataset, a total of 54 groups of pervious concrete specimens were prepared. The experimental program primarily considered three variables: aggregate size, gradation, and target porosity. Diabase aggregate, with properties detailed in Table 1, was used in three size fractions: 2.36–4.75 mm, 4.75–9.5 mm, and 9.5–16 mm. The target porosity was designed at three levels: 15%, 20%, and 25%. For all experimental groups, P.O 42.5 Ordinary Portland Cement was used, and the water-cement ratio (w/c) was held constant at 0.30. The sound absorption performance was evaluated using the impedance tube method on cylindrical specimens (Φ100 mm × 50 mm) (Fig. 2). The average sound absorption coefficient across the full frequency range was calculated according to Eq. (1) to quantify the acoustic performance^21,25^. The detailed experimental design and the corresponding test results are presented in Table 2.

1 $$\alpha =\frac{{{\alpha _1}+\cdot\cdot\cdot{\alpha _n}}}{n}$$

where α represents the average sound absorption coefficient over the full frequency range, α_1_ is the sound absorption coefficient at 250 Hz, and α_n_ is the coefficient at 1600 Hz.

**Table 1:** Physical properties of the diabase aggregate.

Aggregate size (mm)	Apparent density(g/cm^3^)	Compacted bulk density(g/cm^3^)	Stacked porosity (%)	Crush value (%)
2.36 –4.75	2.790	1.538	44.89	13.6
4.75–9.5	2.758	1.555	43.62	9.5
9.5–13.2	2.756	1.548	43.83	9.1
13.2–16	2.756	1.548	43.83	9.1

**Table 2:** Test scheme and results.

Code	Aggregate size (mm)				Porosity	Mean sound absorption coefficient
	2.36–4.75	4.75–9.5	9.5–13.2	13.2–16		
S1	1	0	0	0	18.14	0.338
S2	1	0	0	0	22.92	0.358
S3	1	0	0	0	24.80	0.362
S4	0	1	0	0	18.48	0.247
S5	0	1	0	0	20.11	0.267
S6	0	1	0	0	25.64	0.262
S7	0	0	1	0	19.71	0.236
S8	0	0	1	0	21.28	0.238
S9	0	0	1	0	24.76	0.248
S10	0	0	0	1	17.98	0.221
S11	0	0	0	1	20.81	0.230
S12	0	0	0	1	22.78	0.241
D1	0	0	0.33	0.67	16.08	0.216
D2	0	0	0.33	0.67	18.28	0.220
D3	0	0	0.33	0.67	22.00	0.227
D4	0	0	0.67	0.33	16.56	0.185
D5	0	0	0.67	0.33	21.67	0.190
D6	0	0	0.67	0.33	24.20	0.205
D7	0	0.33	0	0.67	16.32	0.213
D8	0	0.33	0.67	0	15.60	0.219
D9	0	0.33	0.67	0	20.24	0.244
D10	0	0.33	0.67	0	19.12	0.232
D11	0	0.67	0	0.33	17.58	0.229
D12	0	0.67	0.33	0	16.63	0.233
D13	0	0.67	0.33	0	19.23	0.262
D14	0	0.67	0.33	0	26.20	0.234
D15	0.33	0	0	0.67	16.38	0.201
D16	0.33	0	0.67	0	17.33	0.259
D17	0.33	0.67	0	0	19.83	0.276
D18	0.67	0	0	0.33	18.58	0.275
D19	0.67	0	0.33	0	18.09	0.254
D20	0.67	0.33	0	0	19.40	0.304
T1	0.33	0	0.33	0.33	16.54	0.232
T2	0	0.33	0.33	0.33	17.92	0.222
T3	0	0.33	0.33	0.33	22.11	0.239
T4	0	0.33	0.33	0.33	24.95	0.232
T5	0.33	0.33	0.33	0	14.68	0.215
T6	0.33	0.33	0.33	0	19.42	0.245
T7	0.33	0.33	0.33	0	23.43	0.290
T8	0.25	0	0.25	0.5	18.54	0.267
T9	0.33	0.33	0	0.33	15.96	0.238
T10	0.25	0	0.5	0.25	16.81	0.202
T11	0.25	0.25	0.5	0	17.07	0.237
T12	0.25	0.5	0	0.25	16.88	0.203
T13	0.25	0.5	0.25	0	18.06	0.293
F1	0.1	0.15	0.15	0.6	18.99	0.264
F2	0.15	0.15	0.15	0.55	16.72	0.188
F3	0.15	0.15	0.15	0.55	17.98	0.204
F4	0.15	0.15	0.15	0.55	19.29	0.215
F5	0.15	0.15	0.30	0.40	16.00	0.176
F6	0.45	0.15	0.15	0.25	16.55	0.176
F7	0.25	0.25	0.25	0.25	17.04	0.211
F8	0.25	0.25	0.25	0.25	16.39	0.202
F9	0.25	0.25	0.25	0.25	20.48	0.235

#### Data collected from published literature

To augment the scale and improve the diversity of the training dataset, an extensive collection of acoustic performance data was collated from the existing scientific literature. This supplementary dataset, combined with the data generated in this study, formed a comprehensive basis for the subsequent model training. The specific data points extracted from the literature are itemized in Table 3.

**Table 3:** Summary of data from the literature^21,25^.

Code	Aggregate size (mm)				Porosity	Mean sound absorption coefficient
	2.36–4.75	4.75–9.5	9.5–13.2	13.2–16		
1	0.10	0.90	0	0	20.51	0.292
2	0.1	0.9	0	0	17.50	0.274
3	0.1	0.9	0	0	15.00	0.263
4	0.2	0.8	0	0	20.60	0.272
5	0.2	0.8	0	0	16.80	0.262
6	0.20	0.80	0.00	0	13.70	0.254
7	0	0.90	0.10	0	20.50	0.303
8	0	0.90	0.10	0	17.70	0.297
9	0	0.90	0.10	0	15.90	0.277
10	0	0.80	0.2	0	20.50	0.284
11	0	0.8	0.2	0	18.70	0.271
12	0	0.8	0.2	0	16.80	0.262
13	0.1	0.8	0.1	0	20.22	0.286
14	0.1	0.8	0.1	0	17.80	0.274
15	0.10	0.80	0.10	0	15.30	0.267
16	0.10	0.70	0.20	0	19.60	0.274
17	0.10	0.70	0.20	0	17.80	0.263
18	0.10	0.70	0.20	0	15.30	0.257
19	0.15	0.70	0.15	0	19.70	0.297
20	0.15	0.7	0.15	0	17.20	0.28
21	0.15	0.7	0.15	0	15.28	0.261
22	0.2	0.7	0.1	0	20.70	0.305
23	0.2	0.7	0.1	0	17.80	0.297
24	0.20	0.70	0.10	0.00	14.90	0.286
25	0.20	0.60	0.20	0.00	20.90	0.289
26	0.20	0.60	0.20	0.00	17.90	0.281
27	0.20	0.60	0.20	0.00	16.20	0.27
28	1	0	0	0	21.80	0.331
29	1	0	0	0	17.50	0.303
30	1	0	0	0	14.60	0.274
31	0	1	0	0	20.90	0.246
32	0	1	0	0	17.40	0.238
33	0	1	0	0	15.00	0.232
34	0	0	1	0	22.50	0.230
35	0	0	1	0	21.20	0.221
36	0	0	1	0	16.80	0.217

#### Data preparation and augmentation

To mitigate the influence of dimensional disparities among the input parameters, the dataset was first standardized using max normalization, scaling all feature values to a range of [0, 1] according to Eq. (2). Subsequently, to expand the training set and enhance the model’s generalization capability on unseen data, the Mixup data augmentation technique was employed. This method generates virtual samples by linearly interpolating the input data and their corresponding labels, thereby effectively expanding the data space. To completely eliminate the risk of data leakage, the original dataset was first divided into the training set and an independent test set before any augmentation procedure. Mixup augmentation was applied only to the training set, whereas the independent test set was kept unchanged and was never involved in augmentation, hyperparameter tuning, model fitting, or meta-learner training.

2 $$x_{{ij}}^{\prime }=\frac{{{x_{ij}}}}{{\hbox{max} \left\{ {{x_{ij}}} \right\}}} \cdot \cdot \cdot \cdot \cdot \cdot (i=1,2, \cdot \cdot \cdot ,m;j=1,2 \cdot \cdot \cdot ,n)$$

where $\:\mathrm{max}\left\{{x}_{ij}\right\}$ is the maximum value of the corresponding feature.

Regarding the physical realism of the data augmentation, it is important to note that all input features in this study (aggregate size fractions and porosity) have strict physical boundaries (i.e., proportions bounded between 0 and 1). Because the Mixup technique generates virtual samples through convex combinations (linear interpolation), the resulting synthetic samples mathematically represent intermediate, physically plausible mix designs that strictly remain within these physical boundaries. This fundamentally prevents the generation of physically impossible parameters (such as negative aggregate proportions), thereby ensuring the physical realism of the augmented dataset.

### Model training and evaluation

#### Machine learning models

Recognizing that single machine learning models are often constrained by their specific inductive biases when tackling complex engineering problems^28,29^, this study constructed an advanced predictive framework based on Stacking ensemble learning. Stacking effectively mitigates these individual inductive biases by employing a meta-learner to learn an optimal combination of their diverse predictive strategies. The goal is to systematically integrate the strengths of diverse algorithms, thereby breaking through predictive bottlenecks and achieving accurate predictions for the noise reduction performance of pervious concrete. The core of this framework was a two-layer learning architecture with a two-stage training process. In the first stage, the first layer (base-learner layer) involved the parallel training of multiple heterogeneous learners. To strictly prevent information leakage, the dataset was initially partitioned into a training set and a completely independent test set via random sampling prior to any model training. An out-of-fold prediction protocol via internal cross-validation was employed exclusively within the training set to generate predictions, which were subsequently assembled into a new “meta-feature” dataset. The independent test set remained strictly isolated throughout the entire model-selection and Stacking training pipeline, acting effectively as the outer loop in a nested validation scheme, and was exclusively reserved for the final, unbiased evaluation of the model’s generalizability. In the second stage, this meta-feature dataset served as the sole input for the second layer (meta-learner layer) to train a final adjudication model that learns to optimally integrate the information from the underlying models. A diverse portfolio of six base-learners was curated to ensure a wide range of learning strategies. This included a Decision Tree (DT) for interpretability, Multilayer Perceptron (MLP) and Convolutional Neural Networks (CNN) to capture complex non-linear relationships and abstract features, and three high-performance ensemble methods—Random Forest (RF), Gradient Boosting Decision Tree (GBDT), and Extreme Gradient Boosting (XGBoost)—which employ Bagging and Boosting techniques to maximize stability and accuracy^30–39^. A Multiple Linear Regression (MLR) model was chosen as the meta-learner. This selection is rationalized by the physical principle that the material’s bulk properties are a composite function of its microstructural features and by the statistical advantage of using a simple meta-learner to prevent overfitting. The MLR model learns an optimal linear combination of the base-model predictions, effectively fusing their diverse “opinions” to produce a final output that is more robust and generalizable than any of its individual constituents^40^.

Regarding the Convolutional Neural Network (CNN) base-learner, a 1D-CNN architecture was specifically employed to process the tabular dataset. Since the input variables consist of numerical parameters (aggregate size fractions and porosity), the 1D feature vectors for each sample were dynamically reshaped into a three-dimensional tensor format of (Batch_size, Number_of_features, 1) to be compatible with 1D convolutional operations. The customized 1D-CNN architecture comprises an input layer, a 1D-Convolutional layer (configured with 16 filters, a kernel size of 2, and a stride of 1), followed by a ReLU activation function to introduce non-linearity. A 1D-MaxPooling layer was then utilized to down-sample the feature maps. Subsequently, the outputs were flattened into a 1D vector by a Flatten layer and passed through a fully connected layer to generate the final continuous predictions. The primary motivation for incorporating the 1D-CNN on tabular data was to efficiently capture the local sequential interactions among adjacent aggregate size fractions, which naturally exhibit a continuous dimensional distribution. Furthermore, by providing a distinct non-linear feature extraction strategy compared to tree-based models, the 1D-CNN significantly enhanced the model diversity of the base-learner pool. This heterogeneity effectively enriched the meta-features passed to the second layer, thereby improving the overall predictive performance and robustness of the Stacking framework.

#### Hyperparameter optimization and model parameter settings

To ensure the rigor, reproducibility, and optimal performance of the predictive models, a systematic Grid Search technique was employed for the hyperparameter optimization of each base-learner. During this process, a comprehensive search grid was defined for the critical hyperparameters of each algorithm. For instance, the search space explored various hidden layer architectures for the MLP neural network, systematically evaluated combinations of max_depth and n_estimators for the tree-based models (DT, RF, GBDT, and XGBoost), and tested different epochs and batch_size configurations for the CNN model. The Grid Search algorithm exhaustively evaluated all possible parameter combinations within the defined space, utilizing the minimization of Mean Squared Error (MSE) as the objective function to determine the best configurations. The specific parameter settings of the six independent models, which represent the globally optimal solutions derived from this rigorous search process, are summarized in Table 4.

**Table 4:** The parameter settings of the independent model.

Model	Parameter settings and parameter values
MLP	hidden_layer_sizes=(100, 2), activation=’relu’, solver=’adam’, random_state = 15, alpha = 0.0001, max_iter = 500
DT	splitter=’best’, criterion=’mse’, random_state = 15, max_depth = 5
CNN	activation=’relu’, optimizer=’adam’, loss=’mse’, epochs = 500, batch_size = 10
RF	n_estimators = 100, criterion=’mse’, random_state = 15, max_depth = 5
GBDT	n_estimators = 100, learning_rate = 0.1, random_state = 15
XGboost	n_estimators = 100, learning_rate = 0.05, random_state = 15, max_depth = 3

#### Model evaluation method

For a comprehensive performance assessment of the predictive models, four statistical metrics were employed: the Coefficient of Determination (R^2^), Mean Absolute Error (MAE), Root Mean Square Error (RMSE), and Mean Absolute Percentage Error (MAPE)^41,42^. The R^2^ value provides a measure of the model’s goodness of fit, with values closer to 1.0 indicating a more effective model. The MAE represents the mean absolute discrepancy between predicted and true values. The RMSE is particularly sensitive to significant outliers and heavily penalizes large variance errors. The MAPE expresses the prediction accuracy as an intuitive relative percentage. Lower values for MAE, RMSE, and MAPE are indicative of superior model accuracy. These metrics are mathematically defined by the following equations:

3 $$MAE=\frac{1}{n}\sum\limits_{{i=1}}^{n} {\left| {({y_i} - {{\hat {y}}_i})} \right|}$$

4 $${R^2}=1 - \frac{{\sum\nolimits_{{i=1}}^{n} {{{({y_i} - {{\hat {y}}_i})}^2}} }}{{\sum\nolimits_{{i=1}}^{n} {{{({y_i} - {{\bar {y}}_i})}^2}} }}$$

5 $$RMSE=\sqrt {\frac{1}{n}\sum\limits_{{i=1}}^{n} {{{({y_i} - {{\hat {y}}_i})}^2}} }$$

6 $$MAPE=\frac{1}{n}\sum\limits_{{i=1}}^{n} {\left| {\frac{{{y_i} - {{\hat {y}}_i}}}{{{y_i}}}} \right|} \times 100\%$$

where n is the number of samples in the test set, y_i_ and ${{\hat {\rm y}}_{\rm i}}$ are the actual and predicted values for the i-th sample, respectively, and ${\bar {\rm y}}$ is the mean of the actual values in the test set.

To ensure statistical rigor and evaluate the robustness of the constructed models, a systematic stability analysis was integrated into the evaluation framework. Specifically, feature importance analysis was employed to verify the physical significance of the inputs. The Shapiro-Wilk test was conducted on prediction residuals to evaluate model assumptions and potential overfitting. Additionally, a non-parametric Bootstrap analysis was implemented to establish the 95% confidence interval (CI) for prediction errors, explicitly quantifying model uncertainty and stability.

### Stacking-NSGA-II framework for multi-objective optimization

**Fig. 3:**
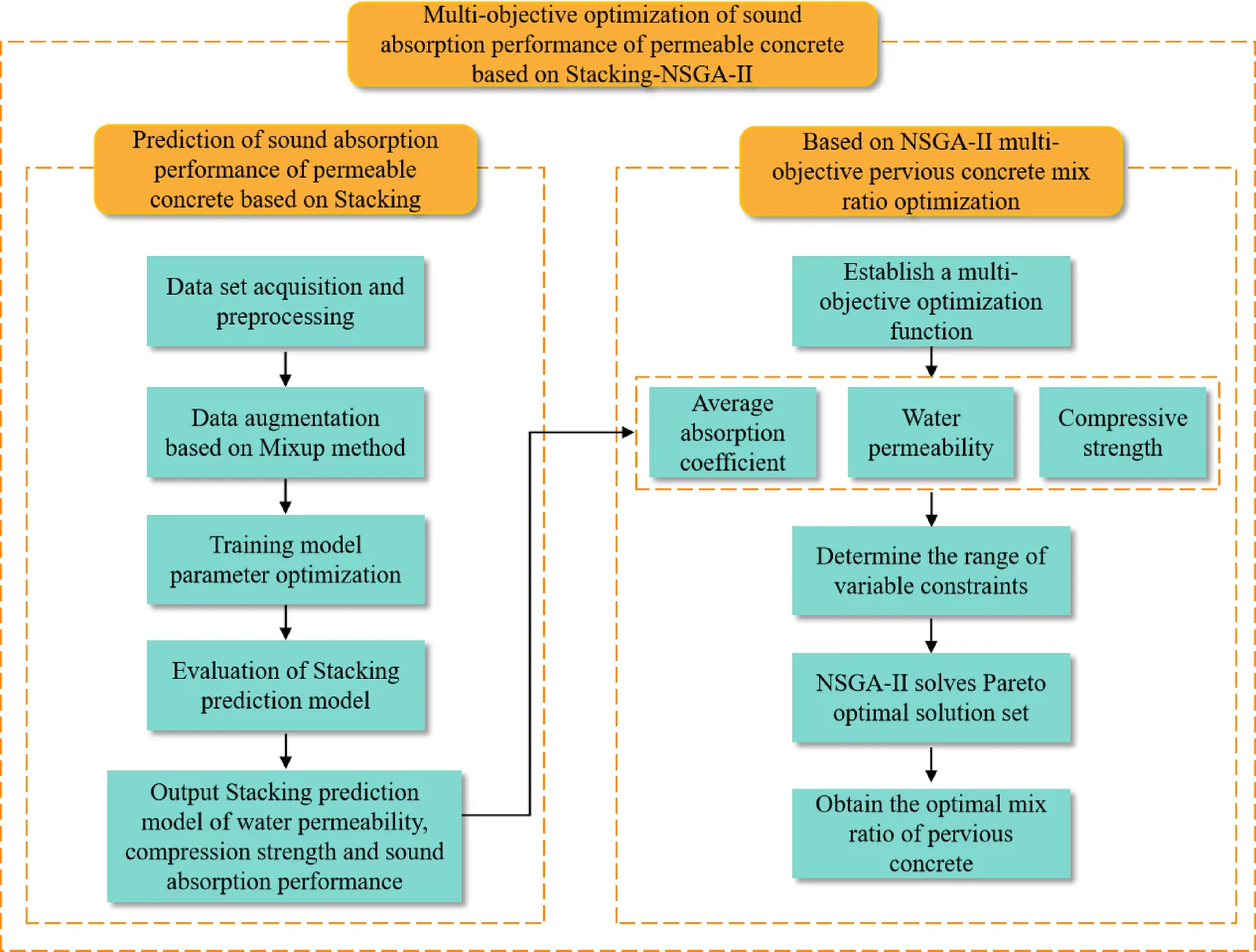
Flowchart of the stacking-NSGA-II optimization algorithm.

The Non-dominated Sorting Genetic Algorithm II (NSGA − II) is a computationally efficient multi-objective evolutionary algorithm that utilizes a crowding distance metric for solution sorting. Despite its widespread application due to operational simplicity and robust adaptability, NSGA − II is susceptible to premature convergence to local optima and can yield a non-uniform distribution of solutions along the Pareto front. To address these limitations, this study proposes an enhanced NSGA − II for the multi-objective optimization of water permeability, compressive strength and sound absorption coefficient in pervious concrete (Fig. 3). The proposed modifications encompass three primary strategies: initializing the population via uniform design, implementing an improved selection operator, and employing a dynamic crowding distance adjustment mechanism. These enhancements are intended to generate a Pareto optimal set with superior convergence and diversity. The specific algorithmic parameters are detailed in Table 5.

**Table 5:** Parameter setting of NSGA-II algorithm.

Parameter name	Objective function	Decision variable	Population size	Crossover probability	Mutation probability	Evolutionary generations
Parameter value or number	2	4	100	0.3	0.9	400

## Results and discussion

### Effect of aggregate gradation on sound absorption performance

**Fig. 4:**
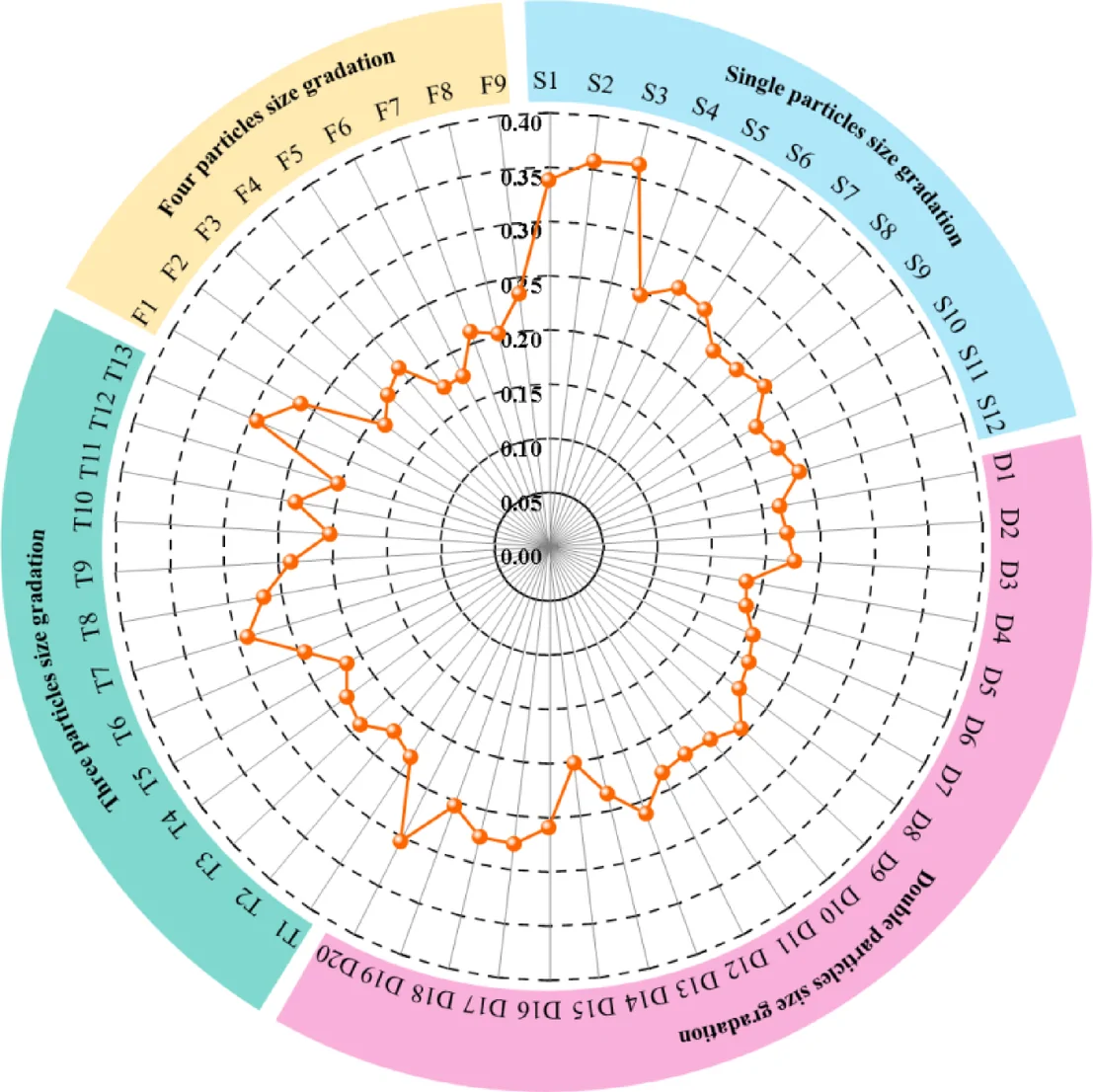
Effect of aggregate gradation on sound absorption performance.

The experimental data analysis (Fig. 4) revealed that aggregate gradation was a primary determinant of the acoustic performance of pervious concrete, with a substantial performance difference observed between the optimal design (S3, 0.362) and the poorest design (D4, 0.185). Specifically, the sound absorption coefficient of the optimal design S3 exhibits a relative increase of 95.7% compared to the poorest design D4. This highlights that proper gradation design is crucial. While both porosity and aggregate gradation influence sound absorption—with performance generally improving with porosity—the aggregate size was identified as the more dominant factor. Specifically, gap-graded mixes with two or three aggregate sizes were found to outperform the conventional single-sized 4.75–9.5 mm material. The highest sound absorption coefficient (0.362) was achieved with a single small aggregate size (2.36–4.75 mm), and performance was observed to degrade as the single aggregate size increased.

The predominant influence of smaller aggregates was also evident in mixed-gradation designs. High-performing samples, including D20 (0.304), T7 (0.290), and T13 (0.293), were all characterized by a high proportion of small- and medium-sized particles. This suggests that increasing the fraction of smaller aggregates is an effective strategy for acoustic enhancement. Interestingly, continuously graded mixes incorporating four particle sizes yielded lower overall sound absorption than the best-performing gap-graded mixes. While some continuously graded structures (e.g., the F series) might promote sound dissipation through complex pore networks, the results strongly indicate that carefully optimized gap gradations, rather than continuous gradations or single sizes, represent the most effective approach for maximizing the sound absorption of pervious concrete.

### Comparison between stacking models and independent models

**Fig. 5:**
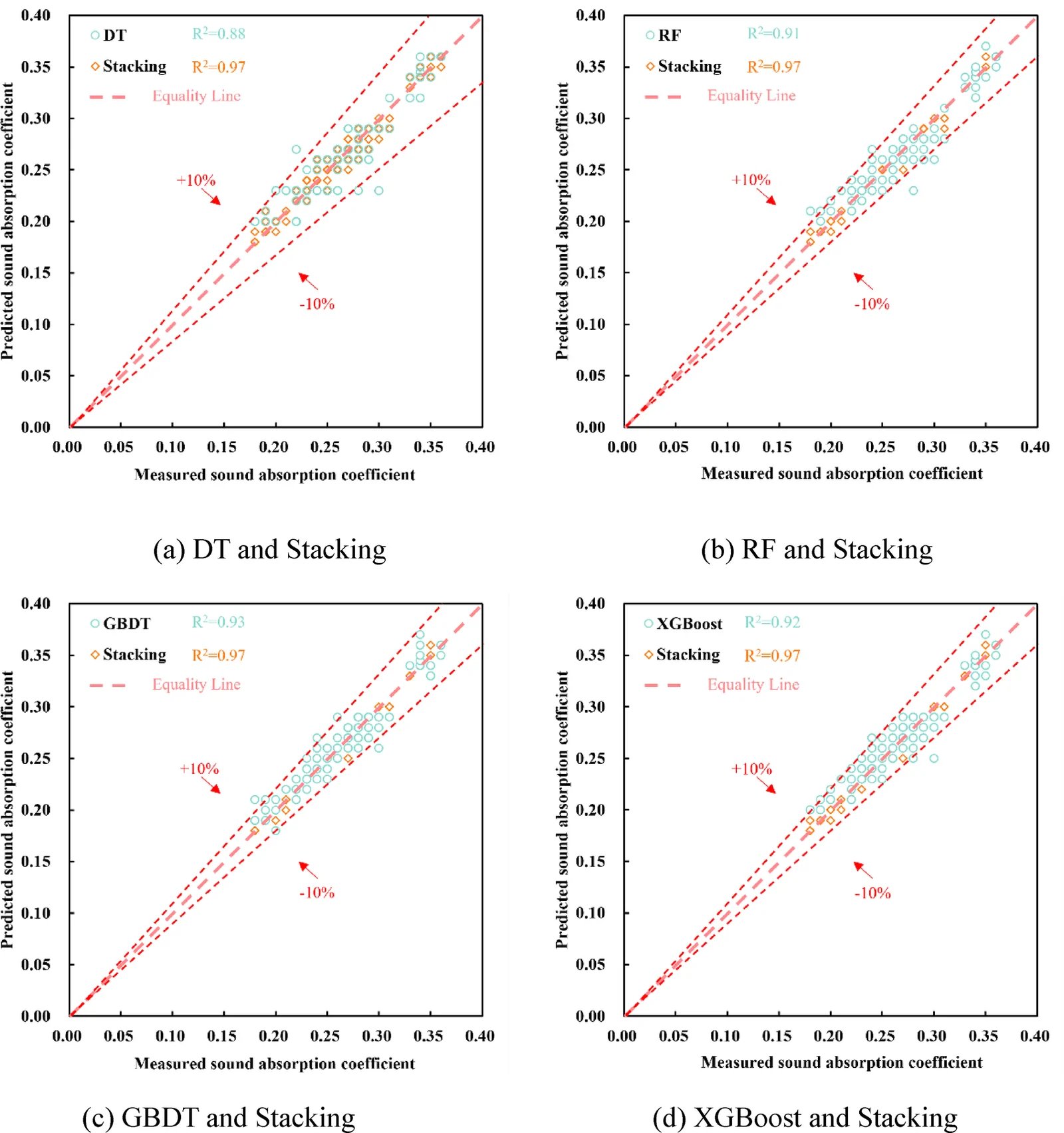

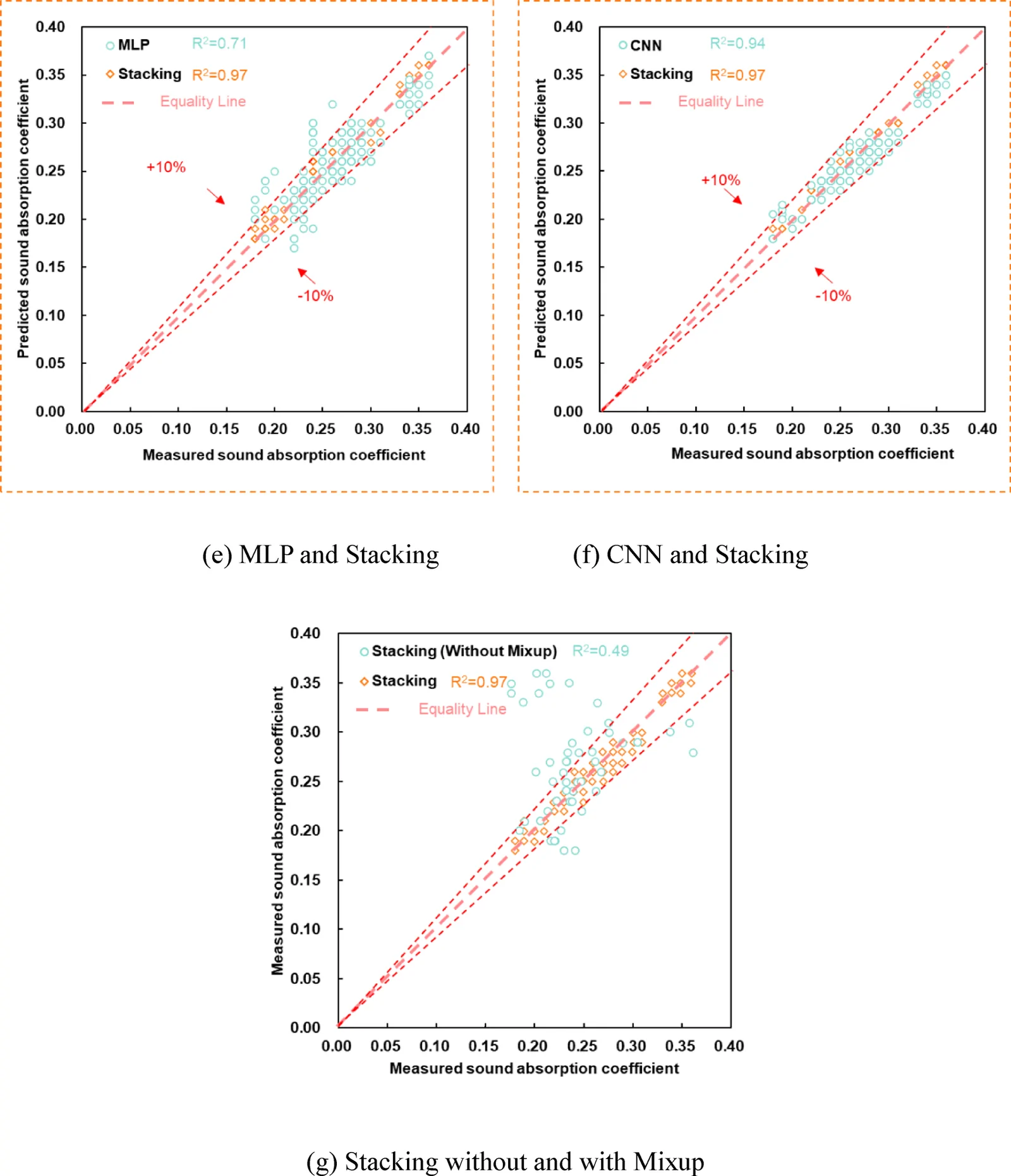
Predictions of sound absorption coefficient made by different models. (**a**) DT and Stacking, (**b**) RF and Stacking, (**c**) GBDT and Stacking, (**d**) XGBoost and Stacking, (**e**) MLP and Stacking, (**f**) CNN and Stacking, (**g**) Stacking without and with Mixup.

To quantitatively and visually compare the predictive performance of the Stacking ensemble model against the baseline single models, a rigorous evaluation was conducted on an independent test set. The scatter plots comparing the predicted versus actual values for each model are presented in Fig. 5.

In Fig. 5, ideal predictions should be tightly distributed around the 45° diagonal line, which represents a perfect match between predicted and actual values. Observation of the results indicates that the scatter plot for the Stacking model exhibits a high degree of linear concentration. The vast majority of data points are closely clustered around the 45° diagonal, and all predictions fall within the predefined ± 10% error band. This phenomenon not only visually confirms the high predictive accuracy of the Stacking model but also reflects its strong generalization ability and stability. In contrast, the performance of the individual baseline models was inconsistent. For example, the predictions from the MLP and DT models show a large number of scattered points that deviate significantly from the ± 10% error band, indicating substantial prediction errors and unsatisfactory performance. From the perspective of key performance metrics, the Stacking model achieved a Coefficient of Determination (R^2^) of 0.97, the highest among all models, further validating its superior predictive capability. The performance advantage of the Stacking model is rooted in its hierarchical learning paradigm: the second-level meta-learner effectively integrates and learns from the output biases of the first-level base-learners while retaining key information from the original dataset, thereby achieving optimization and correction of the final prediction.

Notably, among the baseline models, CNN, XGBoost, and GBDT also demonstrated competitive performance, with R^2^ values of 0.94, 0.93, and 0.92, respectively. However, despite these proximate R^2^ values, the scatter distributions in Fig. 5 reveal a critical difference in their predictive stability compared to the Stacking model. The predictions from the CNN and XGBoost models contain more outliers, with some predictions even exceeding the ± 10% error threshold. This suggests a higher risk of generating large errors when encountering unseen data, indicating that their stability is inferior to that of the Stacking ensemble model.

Furthermore, to definitively verify that the exceptionally high predictive performance is not merely a linear artifact induced by the Mixup augmentation process, a baseline comparative analysis was conducted. The identical Stacking ensemble framework was trained and evaluated exclusively on the entire original unaugmented dataset. The results showed that due to the small sample size, the highly complex ensemble model faced severe learning difficulties, resulting in a significantly lower baseline R^2^ of 0.49 with a highly divergent scatter distribution. This stark contrast strongly indicates that the high performance is not a simple linear fabrication. Instead, Mixup acted as a crucial regularization technique. By filling a reasonable transitional feature space between known samples, it effectively suppressed the overfitting collapse of the model on the small dataset, thereby substantially enhancing the model’s robustness in capturing true non-linear rules.

### Model stability analysis

**Fig. 6:**
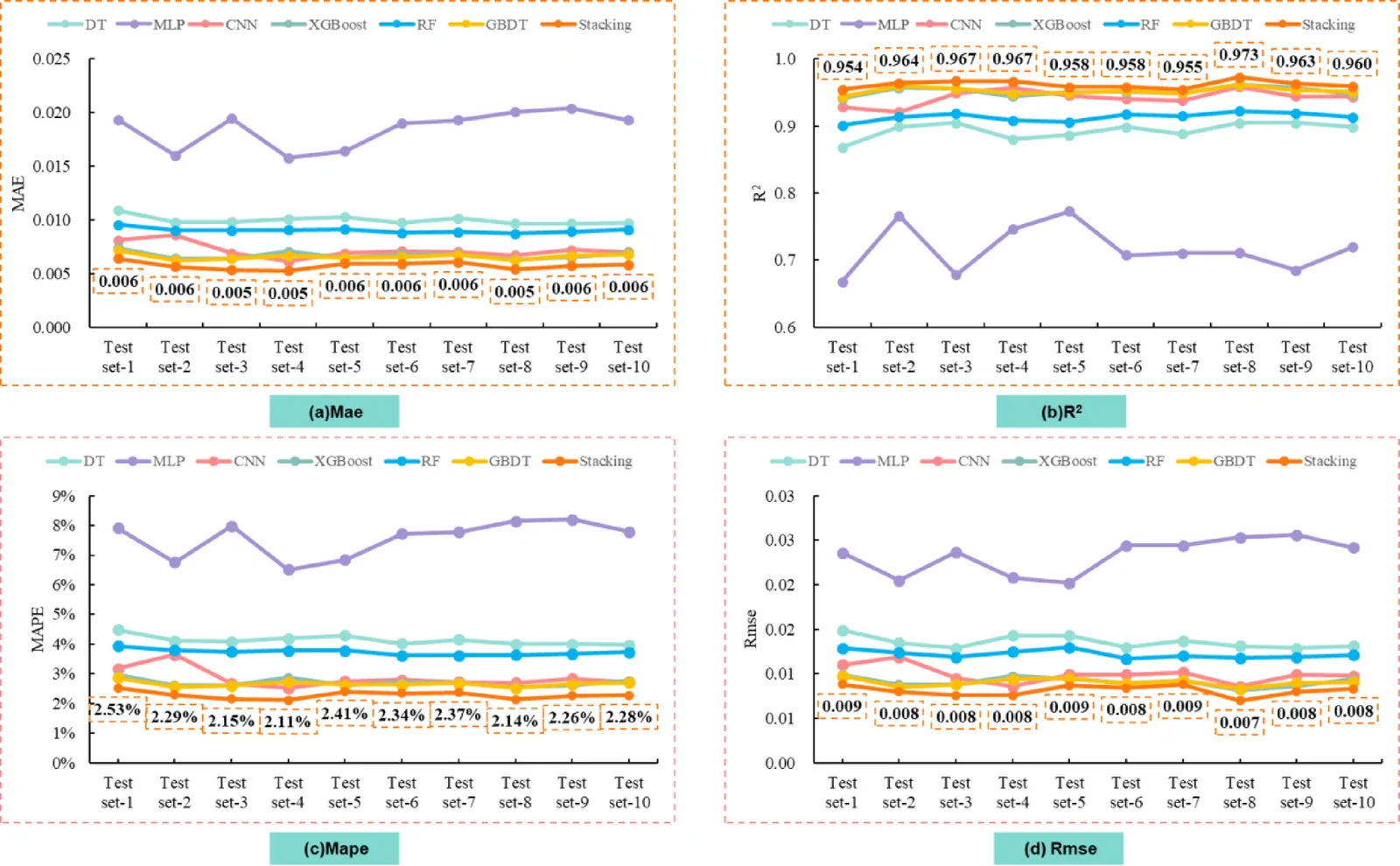
Results of model stability analysis.

In addition to predictive accuracy, model stability—defined as the ability to maintain a high level of performance across different datasets—is a core criterion for evaluating a model’s practical value. To systematically investigate the stability of the proposed models, a validation strategy based on random subset sampling was employed. 10 independent subsets, each containing 100 data samples, were randomly drawn without replacement from the original test set. By evaluating the performance metrics of each model on these subsets, the consistency and robustness of their predictions could be analyzed. The MAE and R^2^ values for each model across the 10 sub-test sets are presented in Fig. 6.

The evaluation results clearly demonstrate that the Stacking ensemble model’s performance is not only accurate but also highly stable. Across all 10 tests, the MAE values for the Stacking model consistently remained at the lowest level, with a minimal fluctuation range (0.005–0.006). Concurrently, its R^2^ values exhibited very little variation (0.954–0.973), staying consistently close to 1 and significantly outperforming all comparative models. In contrast, although advanced single models like GBDT and XGBoost also achieved favorable prediction results, their performance metrics showed greater fluctuation across the different subsets, and their average accuracy never surpassed that of the Stacking model.

Furthermore, the stability and superior quality of the Stacking ensemble are further corroborated by employing the RMSE and MAPE metrics. Across the 10 subsets, the Mean Absolute Percentage Error (MAPE) of the Stacking model remained exceptionally stable at a minimal level (averaging 2.29%), whereas single models exhibited much higher relative error fluctuations. Crucially, comparing RMSE with MAE reveals deeper insights into predictive robustness. Since RMSE heavily penalizes large variance errors, the remarkably narrow numerical gap between the Stacking model’s RMSE (averaging 0.0081) and its MAE across all 10 test sets indicates a highly uniform error distribution devoid of severe outliers. In contrast, base-learners like the MLP neural network exhibited severe ‘rollercoaster’ trends in their RMSE, exposing their vulnerability to random data permutations. This confirms that the Stacking ensemble successfully avoids catastrophic predictive failures, ensuring highly robust engineering reliability.

To further validate statistical rigor, the Shapiro-Wilk test did not reject the normality assumption of the residuals (*p* = 0.6857), indicating no strong evidence of severe non-normal residual behavior. Together with the independent test-set evaluation and Bootstrap uncertainty analysis, this result supports the robustness of the proposed model within the current data scope. Furthermore, a Bootstrap analysis with 1000 resamples was conducted to estimate the 95% confidence interval of the RMSE. The resulting 95% CI was [0.0233, 0.0315], indicating limited uncertainty in the prediction errors under the current dataset. Finally, to enhance interpretability, a permutation feature importance analysis was conducted on the independent test set. The results showed that the 13.2–16.0 mm aggregate fraction, 2.36–4.75 mm aggregate fraction, and porosity were the most influential variables, which is consistent with the physical mechanisms governing pore connectivity and acoustic energy dissipation.

### Multi-objective optimization and verification

#### Multi-objective optimization strategy and results

A typical trade-off relationship exists among the permeability, compressive strength, and sound absorption performance of pervious concrete. Generally, permeability and compressive strength are negatively correlated, and the balance between these two properties also affects the sound absorption performance. Therefore, designing an effective optimization strategy to synergistically enhance these three properties is of critical importance.

**Fig. 7:**
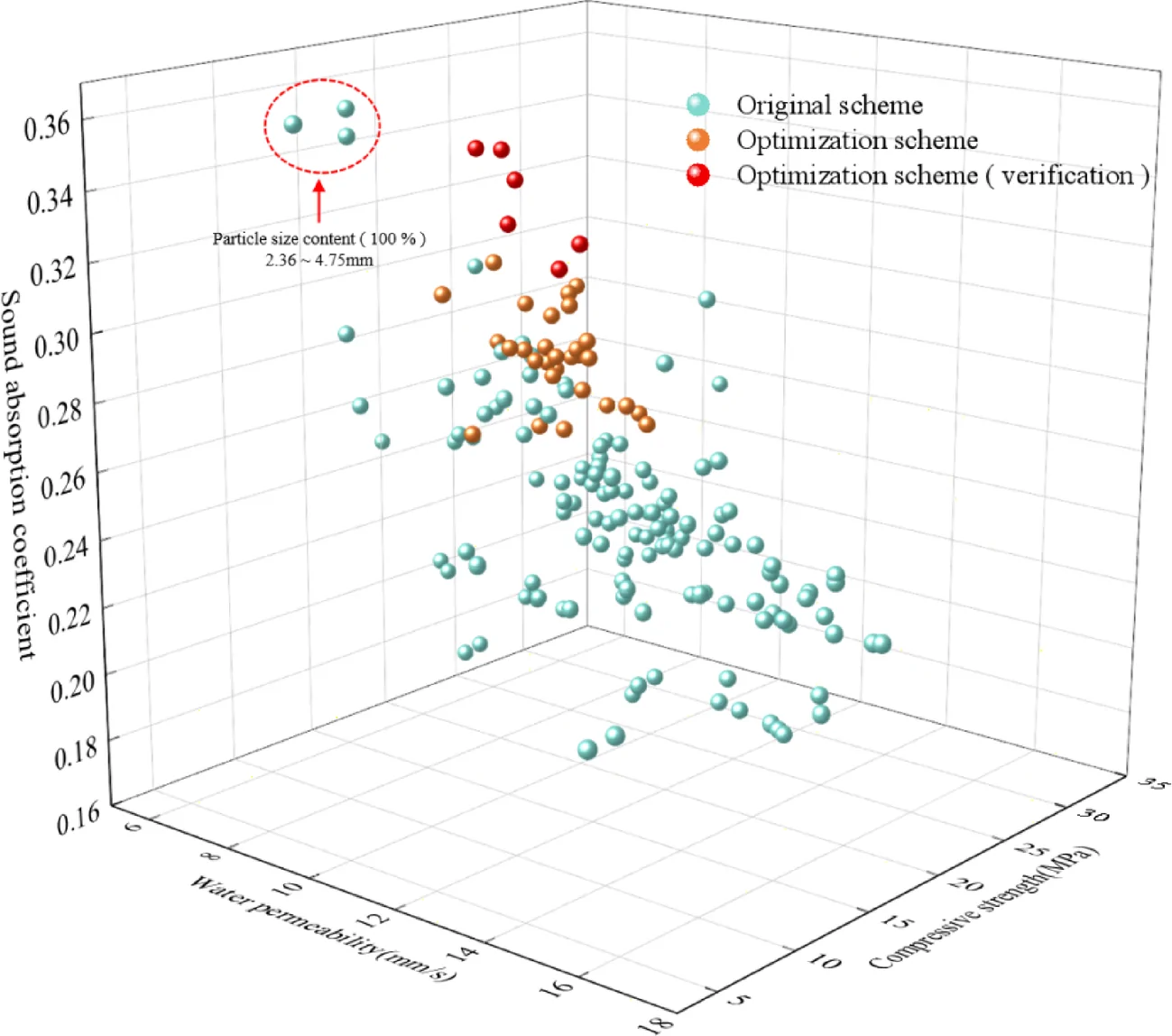
Pareto front of optimal mix proportion combinations.

This study aims to propose an optimization method that maximizes sound absorption performance while ensuring that the permeability coefficient (5–11 mm/s) and compressive strength (15–26 MPa) meet engineering requirements. Therefore, the constraint boundaries for the mix proportions were first determined based on established standards and practical engineering experience. Subsequently, the NSGA-II multi-objective optimization algorithm was employed for a global search. The optimization was executed in a Python environment with the following key algorithm parameters: a crossover operator of 0.3, a mutation operator of 0.9, a population size of 100, and both the maximum and stopping generations set to 400. During the iterative optimization process, permeability, compressive strength, and sound absorption performance were used as the objective functions to find the optimal mix proportion combination. After 400 iterations, the algorithm converged and generated the Pareto front shown in Fig. 7, which clearly illustrates the trade-offs among the different performance targets.

Ultimately, the optimization process yielded 43 sets of mix proportion solutions that satisfied all predefined constraints. As depicted in Fig. 7, compared to the original data, the optimized results not only meet the design requirements for permeability and strength but also exhibit significantly enhanced sound absorption performance. This outcome indicated that the optimization strategy adopted in this study can effectively resolve the conflicts among the multiple properties of pervious concrete and achieve a substantial improvement in its overall performance.

#### Experimental validation of optimization results

To validate the accuracy and reliability of the constructed Stacking-NSGA-II multi-objective optimization model, a systematic experimental validation was conducted. First, the six mix proportion designs with the best sound absorption performance were selected from the Pareto front solution set (Fig. 7), with their specific parameters detailed in Table 6. Subsequently, corresponding pervious concrete specimens were prepared based on these optimized designs. The permeability coefficient, compressive strength, and average sound absorption coefficient of these specimens were then tested in strict accordance with standard experimental methods.

**Fig. 8:**
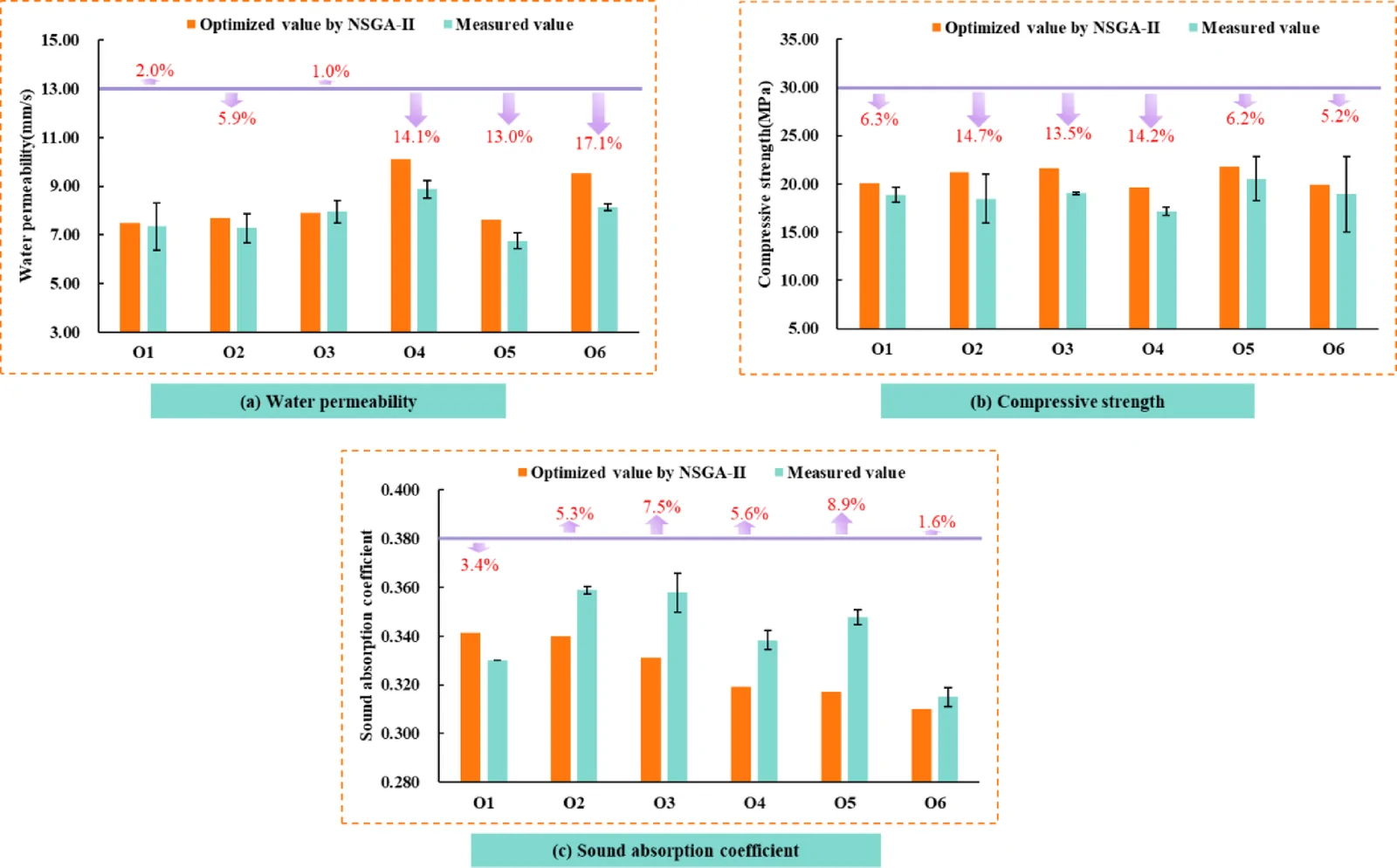
Comparison of predicted and experimental results.

Figure 8 provides a visual comparison between the predicted values and the experimentally measured values. The results show a high degree of consistency between the measured performance of the six specimen groups and the model’s theoretical predictions. Specifically, the maximum relative errors were 17.1% for the permeability coefficient, 14.7% for compressive strength, and only 8.9% for the core optimization target—the average sound absorption coefficient. Notably, the prediction accuracy for sound absorption performance was the highest, which directly demonstrates the effectiveness and high fidelity of the optimization model in enhancing the material’s acoustic capabilities. The slightly higher errors for permeability and compressive strength are inferred to stem from unavoidable random factors during the experimental process. These may include minor fluctuations in aggregate gradation, variations in the vibration and compaction process, and slight differences in curing conditions, all of which can influence the final pore structure and mechanical properties.

In conclusion, the minor discrepancies between the theoretical predictions and the experimental results are within an acceptable range. These validation results fully demonstrate that the multi-objective optimization strategy proposed in this paper is not only feasible but also possesses excellent accuracy and reliability.

**Table 6:** Optimized mix proportions selected for experimental validation.

Code		1	2	3	4	5	6
Aggregate size (mm)	2.36–4.75	0.9	0.882	0.829	0.777	0.843	0.764
	4.75–9.50	0	0	0	0.226	0	0.174
	9.50–13.2	0	0.062	0	0	0	0.054
	13.2–16.0	0.01	0.064	0.171	0	0.16	0
Porosity		20.885	20.723	21.388	19.943	19.745	17.745
Sound absorption coefficient		0.341	0.34	0.331	0.319	0.317	0.31
Permeability (mm/s)		7.494	7.704	7.888	10.121	7.635	9.532
Compressive strength (MPa)		20.058	21.177	21.573	19.6	21.777	19.903

#### Analysis of optimization result

**Fig. 9:**
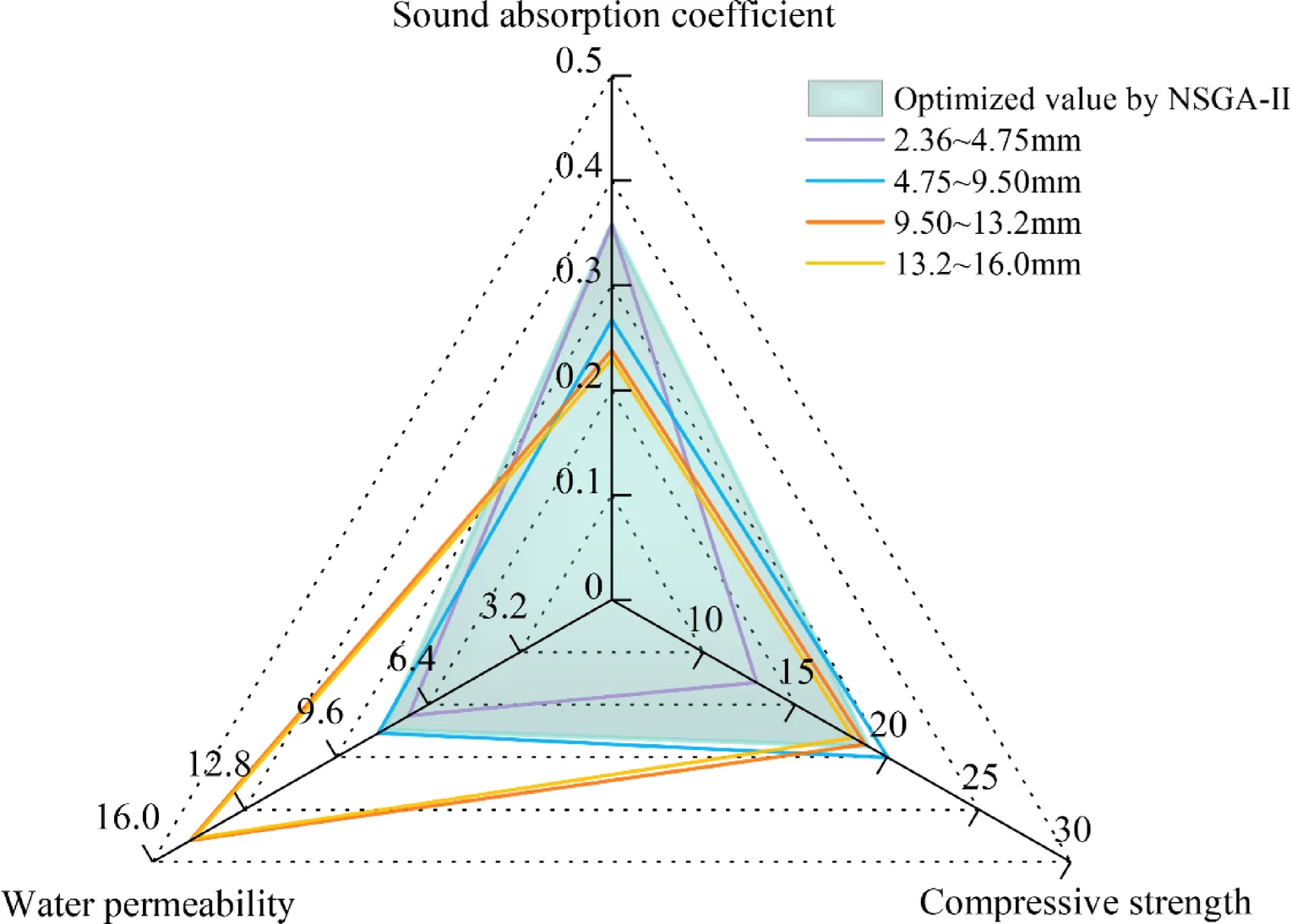
Performance comparison of optimized and conventional mix designs.

Engineering practice and existing research indicate that using single-sized aggregate, particularly in the 4.75 mm to 9.5 mm range, is a conventional and effective method for preparing pervious concrete with balanced permeability and mechanical properties. Therefore, specimens made with single-sized aggregates were selected as the benchmark in this study to evaluate the comprehensive performance advantages of the multi-objective optimization solutions. Based on the preceding experimental validation and considering factors of engineering applicability such as cost-effectiveness and simplicity of the mix design, the O3 solution was chosen from the six optimized candidates as the final recommended mix proportion. Figure 9 presents a detailed performance comparison between the O3 solution and pervious concrete made with four different single-sized aggregates (2.36–4.75 mm, 4.75–9.5 mm, 9.5–13.2 mm, and 13.2–16 mm).

The comparative analysis (Fig. 9) reveals the significant comprehensive performance advantage of the O3 solution, the core of which lies in achieving an “optimal balance” among key properties and overcoming the performance shortcomings commonly found in single-sized aggregate designs. Specifically, the permeability coefficient of the O3 solution reached as high as 7.89 mm/s. Although this value is slightly lower than that of the large-aggregate groups (9.5–13.2 mm and 13.2–16 mm), it surpasses the conventional 2.36–4.75 mm and 4.75–9.5 mm groups and fully satisfies engineering requirements. The compressive strength of the O3 solution is comparable to that of the mechanically superior 4.75–9.5 mm benchmark group while being significantly higher than all other single-sized aggregate groups, ensuring structural load-bearing capacity and durability. Most critically, the sound absorption performance of the O3 solution not only substantially exceeds that of the medium- and large-aggregate groups but is also slightly superior to the small-aggregate (2.36–4.75 mm) group, which is typically best for sound absorption.

In conclusion, conventional single-sized aggregate designs struggle to simultaneously achieve high performance in permeability, mechanical strength, and sound absorption. The O3 solution, obtained through the enhanced NSGA-II algorithm proposed in this study, successfully overcomes this performance constraint. It achieves a synergistic enhancement of permeability and sound absorption while ensuring that critical mechanical performance is not compromised. This result re-confirms that the proposed optimization method is an advanced and effective technical approach for developing multifunctional, high-performance pervious concrete.

## Conclusion

In this study, a sound absorption prediction model was constructed and combined with existing models for compressive strength and permeability to serve as the fitness functions for the NSGA-II algorithm, enabling multi-objective optimization. The optimization results were subsequently validated through experiments, which confirmed the accuracy and reliability of the proposed method. The main conclusions are as follows:

1. Aggregate gradation has a significant impact on the sound absorption performance of pervious concrete. The optimal gradation S3 achieved a sound absorption coefficient of 0.362, representing a 95.7% improvement compared with the poorest gradation D4 with a coefficient of 0.185. The relative improvement was calculated as (0.362 − 0.185) / 0.185 × 100% = 95.7%.
2. Compared to six individual baseline models, the Stacking model achieved the highest coefficient of determination ($R^2$ = 0.97) and exhibited the minimal performance fluctuation in random subset tests, demonstrating superior accuracy and stability.
3. The proposed multi-objective optimization framework effectively resolved the intrinsic conflicts and trade-offs among the three key indicators of pervious concrete: permeability, compressive strength, and sound absorption. This was confirmed by the high degree of consistency between theoretical predictions and experimental measurements, with the error for the core metric (sound absorption coefficient) being only 8.9%.
4. The optimized mix proportion solution (represented by O3) satisfied engineering requirements for permeability (7.89 mm/s) and achieved a compressive strength comparable to the mechanically superior benchmark group (single-sized 4.75–9.5 mm). Most notably, its sound absorption performance surpassed all single-sized aggregate groups and was even slightly superior to the small-aggregate (2.36–4.75 mm) group.

In summary, this study advances the current state of the art by establishing a data-driven optimization paradigm that effectively balances the conflicting properties of pervious concrete. From an engineering perspective, this method provides a reliable design tool for low-noise pavements in sponge city applications. Future research will focus on predicting full absorption spectra to enable more precise, band-specific acoustic tuning.

## Data Availability

The custom code used for image processing and data analysis during the current study is available from the corresponding author on reasonable request.
